# Supplementary material for: Human CCL3L1 copy number variation, gene expression, and the role of the CCL3L1-CCR5 axis in lung function
Source: Wellcome Open Res. 2018 Apr 30;3:13. Originally published 2018 Feb 21. [Version 2] doi: 10.12688/wellcomeopenres.13902.2 (PMC5883389; doi:10.12688/wellcomeopenres.13902.2)
Supplement: Peer review history

# Peer review: Referee response for version 1

**DOI:** 10.21956/wellcomeopenres.15113.r32277

**Published:** 2018-04-03

**Authors:** Peter H. Sudmant

**Affiliations:** 1 Department of Biology, Massachusetts Institute of Technology (MIT), Cambridge, MA, USA

In this manuscript Adewoye, Shrine, and colleagues report on genotyping of 5000 individuals from the UK Biobank for CCL3L1 copy number and CCR5d32 genotype using the paralog ratio test and imputation, respectively. These loci are of biomedical interest due to their role in MIP-1alpha signaling and inflammatory response pathways. The manuscript confirms the association of CCL3L1 copy number with mRNA levels using 1000 Genomes Individuals and goes on to test if CCL3L1 or CCR5d32 genotypes correlate with high/low FEV in heavy smokers from UK Biobank. No association is found with FEV and CCL3L1/CCR5d32 genotype. Overall the manuscript is very clear and well written and represents an important and useful research contribution. 

- In Table 1, it would be helpful to have the footnote (“Values are Mean / SD (range)”) in the legend instead of as a footnote.
- What criteria was used to throw out the third inconsistent PRT? It says “significantly lower than the other two PRT values,” though it’s not really clear what this means. 
- I don’t understand why the authors report the mRNA ratio between CCL3L1 and CCL3 (Figure 3C). What is the significance of this? The variance decreases, but, it’s not clear to me why? If the authors are trying to show the impact of CCL3L1 mRNA levels on CCL3 levels it would be better shown by a scatter plot of CCL3L1 mRNA vs CCL3 mRNA
- In the methods it says that Cufflinks was used to estimate FPKMs, but in the main text it states “We used the specific sequence changes between *CCL3L* and *CCL3* to distinguish transcripts from either gene.” Was an additional procedure beyond Cufflinks used here? It is not clear. If so, this procedure should be described in the methods.
- The authors report figure 3 before figure 2c. It would be helpful to the reader to just mention 2c in passing at least before figure 3. 
- In Figure 3 it would be nice to have the p-values and R^2 values in addition to the slopes of the lines reported on the graphs. The slopes are interesting as they appear to be <1, indicating that there isn’t quite a perfect relationship between increased copy and increased mRNA dosage. 

I have read this submission. I believe that I have an appropriate level of expertise to confirm that it is of an acceptable scientific standard.

# Response

**Published:** 2018-04-25

**Authors:** Ed Hollox

**Affiliations:** Genetics, Leicester, UK

We thank the reviewer for his comments. In response to his original points (which are highlighted in italics):

*In Table 1, it would be helpful to have the footnote (“Values are Mean / SD (range)”) in the legend instead of as a footnote.*

 In this journal’s format, table legends have been converted to footnotes.

*What criteria was used to throw out the third inconsistent PRT? It says “significantly lower than the other two PRT values,” though it’s not really clear what this means.*

 In some samples the LTR61A test amplicon from the LTR61A PRT was split into two separate peaks, with only one peak called by the Genemapper software. This may be due to a small indel in same samples. This resulted in the LTR61A value being significantly below that expected given the values for the other two PRTs. These samples were identified by manual inspection of the capillary electropherograms, and for these samples only CCL3 and CCL4 PRT results were used.

*In the methods it says that Cufflinks was used to estimate FPKMs, but in the main text it states “We used the specific sequence changes between CCL3L and CCL3 to distinguish transcripts from either gene.” Was an additional procedure beyond Cufflinks used here? It is not clear. If so, this procedure should be described in the methods.*

 No additional procedure was used beyond Cufflinks. Because the CCL3L and CCL3 transcripts can be distinguished by multiple specific sequence changes, they are mapped to CCL3L and CCL3 specifically using Cufflinks. We verified this by examining the alignments manually using the IGV browser.

*The authors report figure 3 before figure 2c. It would be helpful to the reader to just mention 2c in passing at least before figure 3.*

 Agreed, this has been modified.

*In Figure 3 it would be nice to have the p-values and R^2 values in addition to the slopes of the lines reported on the graphs. The slopes are interesting as they appear to be <1, indicating that there isn’t quite a perfect relationship between increased copy and increased mRNA dosage.*

 We disagree with placing these values on the graph as the relationships are clear, we wanted to minimise excess information on the graph, and these numbers are given in the text. We have added the value of the slope to the text. We agree that there is not a perfect relationship between copy number and mRNA levels, and this is presumably due to other factors that influence transcription levels of these genes.

# Peer review: Referee response for version 1

**DOI:** 10.21956/wellcomeopenres.15113.r31105

**Published:** 2018-03-08

**Authors:** John A. L. Armour

**Affiliations:** 1 School of Life Sciences, Medical School, Queen's Medical Centre, University of Nottingham, Nottingham, UK

This exploration of association between lung function and CCL3L1 copy number and CCR5 deletion status has the interesting motivation that if the CCR5 signalling axis is associated with lung function, CCR5 antagonists might be repurposed for treatment of respiratory disease. I thought that the methods adopted were well-powered, the analyses appropriate and the conclusions drawn well justified. The mix of information from different cohorts and different genetic analyses was carefully handled to allow powerful conclusions to be derived from high-throughput data sources, while at the same time ensuring that genotypes and copy numbers were properly substantiated by direct DNA typing.

 CNVrd2 does a good job of extracting copy number states for CCL3L1/CCL4L1 from 1000 Genomes Project sequence data, and the concordance in directly typed samples (Figure 1) is impressive. The clustering of the results from the UK Biobank samples is also very clear, validating the precision and accuracy of the Gaussian mixture models extracted by CNVtools.

 For the gene expression analyses at the RNA level I would agree with the conclusions stated in the first paragraph of the Discussion, that although the transcription level for CCL3L1 is much lower than for CCL3, the greater biological effect of each CCL3L1 protein molecule is likely to mean that the variation attributable to CNV has genuine function effect.

 Overall, I think this is an interesting study carried out to a high standard of technical accuracy and robustness, and I have no suggestions for its improvement. One suggestion I offer the authors for their consideration is the possibility of using local SNPs to impute CCL3L1 CN from Biobank data. Clearly, any imputation of a multiallelic CNV from diallelic SNPs is likely to have limited power, but even incomplete imputation of CCL3L1 CN from SNP data alone may unlock power by allowing analysis of many more samples. The CCL3L1 CNV has no simple relationships with flanking SNPs, but the availability of CN measurements for 5000 Biobank samples may allow the exploration of SNP-CNV phasing, to ask whether phased haplotypes could form the basis of partial SNP-CNV imputation, or whether there is essentially complete linkage equilibrium between CNV and flanking SNPs. We have had some (unpublished) successes with the alpha-defensins using MOCSphaser (Kato et al. 2008, Bioinformatics ^1^), and it could be useful to ask whether samples typed only at SNPs could be included in association analyses. Having said that, our own experience has been that the UK Biobank Axiom chip has sparse representation of SNPs around the amylase CNV, and it may be that the density of local SNPs near CCL3L1 may also be low because of the difficulties caused by local paralogy and CNV.

 Minor typos (it would be much easier to specify these if the manuscript had line numbers):

 Introduction, paragraph 3 has “In humans, Mip1apha…”

 There is variation throughout in the name given to the 32bp deletion in CCR5, and in particular whether the CCR5 component is italicised, and whether d or del is used.

 In the Methods, section “CCL3L1 copy number estimation …”, I wasn’t sure what the intended meaning was for the sentence beginning “A prior information…”. Unless “an information” is a technical term I don’t know, presumably this should either be “Prior information…” or “A priori information…”. The following sentence should read “was fed into a Bayesian model…”.

I have read this submission. I believe that I have an appropriate level of expertise to confirm that it is of an acceptable scientific standard.

## References

[1] KatoMNakamuraYTsunodaT: MOCSphaser: a haplotype inference tool from a mixture of copy number variation and single nucleotide polymorphism data. *Bioinformatics*.2008;24(14) : 10.1093/bioinformatics/btn242 1645-6 10.1093/bioinformatics/btn242 18492685PMC2638261

# Response

**Published:** 2018-04-25

**Authors:** Ed Hollox

**Affiliations:** Genetics, Leicester, UK

We thank the reviewer for his comments and helpful suggestions. We have corrected the typographical errors in our revised manuscript.
